# Pathogenesis of intestinal *Pseudomonas aeruginosa* infection in patients with cancer

**DOI:** 10.3389/fcimb.2013.00115

**Published:** 2014-01-07

**Authors:** Panayiota Markou, Yiorgos Apidianakis

**Affiliations:** Department of Biological Sciences, University of CyprusNicosia, Cyprus

**Keywords:** inflammation, tumour, *Drosophila*, human, gut, epithelial damage, regeneration

In 1882, Robert Koch suggested four postulates that establish causation between an infectious agent and a particular disease: (1) the infectious organism must be found in abundance in all diseased, but not in healthy, organisms, (2) the infectious organism must be isolated from the diseased host and grown in culture, (3) the disease must be reproduced when the cultured organism is introduced into a healthy organism and (4) the same organism must be reisolated from the experimentally diseased host (Tabrah, 2011; Breitschwerdt et al., 2013). In Figure 1 we suggest an adaptation to the original postulates of Koch to be used as a framework to assess the causation between intestinal *Pseudomonas aeruginosa* and intestinal disease in patients with cancer. In the following sections we describe the prevalence of *P. aeruginosa* in cancer and the immunosuppressive and stress-inducing conditions of cancer that facilitate the growth, dissemination and virulence of intestinal *P. aeruginosa*. In addition, we describe work showing that *P. aeruginosa* promotes intestinal epithelium cancer-related phenotypes when introduced in tumor prone model hosts.

**Figure 1:**
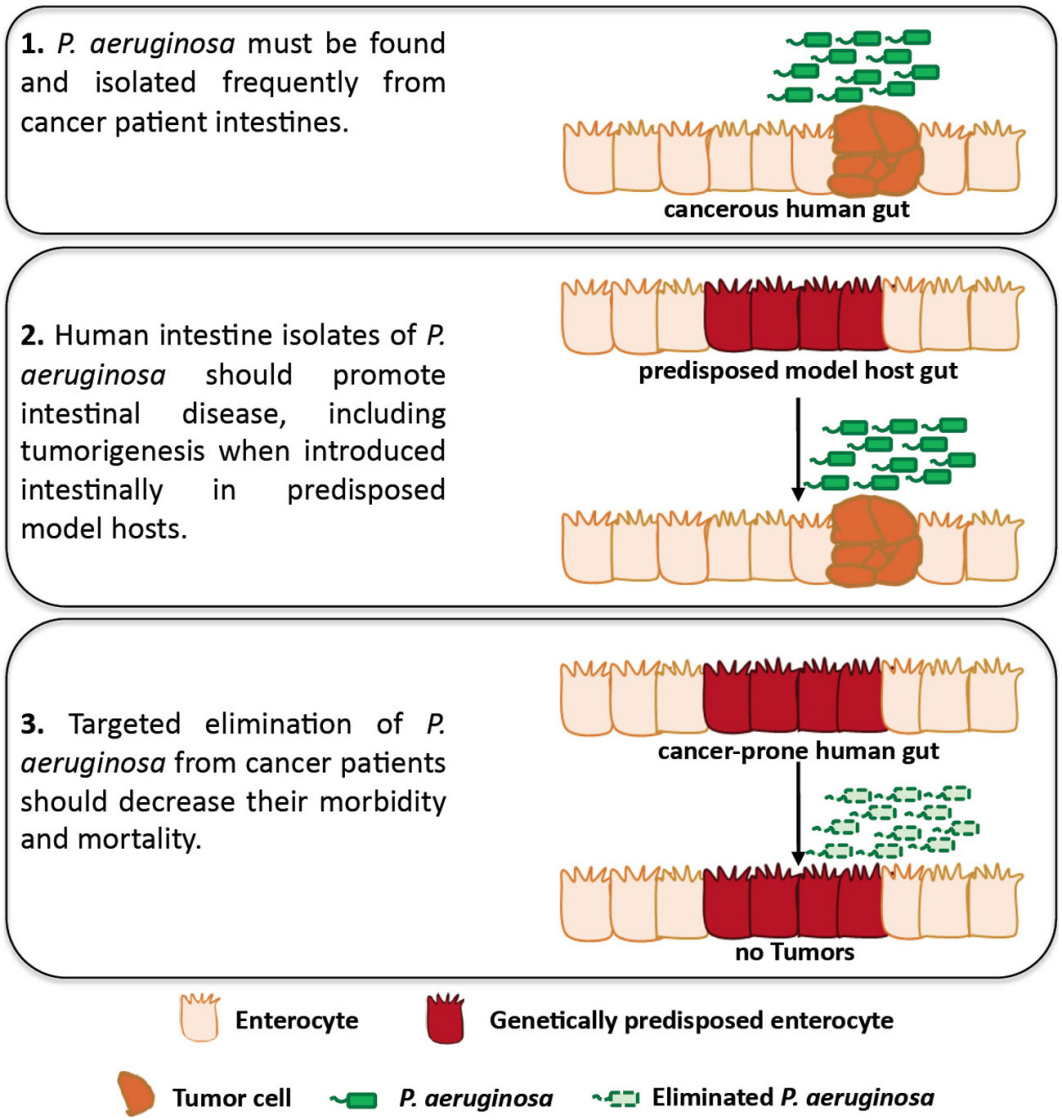
**An adaptation of Koch's postulates assessing causation between intestinal *P. aeruginosa* and disease in patients with cancer.** Studies using *Drosophila* and mammalian hosts may assess the role of *P. aeruginosa* in facilitating intestinal disease, including intestinal *P. aeruginosa* growth, dissemination, virulence and tumorigenesis, in predisposed hosts. In addition, clinical studies can be designed to assess the presence of *P. aeruginosa* in cancer patients and its role in intestinal disease, including tumorigenesis for which clinical data are lacking.

## Cancer and other immunosuppressive conditions promote the prevalence of *P. aeruginosa*

Bacteremia is a major cause of life-threatening complications in patients with cancer, especially those who receive anticancer chemotherapy. Cancer patients are more vulnerable to invasive infection, due to ulcerative lesions in mucosal surfaces and immune suppression secondary to chemotherapy (Safdar and Armstrong, 2001). Many studies associate bloodstream infections in cancer patients with Gram-negative bacteria (Oliveira et al., 2007; Bos et al., 2013; Montassier et al., 2013).

*P. aeruginosa* is a Gram-negative opportunistic bacterium that causes various infections. Common community-acquired infections with *P. aeruginosa* are skin and soft tissue infections, ulcerative keratitis and otitis externa, while hospital-acquired infections include bloodstream infections, pneumonias and urinary tract infections (Driscoll et al., 2007). Infections may be associated with a high rate of morbidity and mortality in immunocompromised hosts, such as those suffering from chemotherapy-induced neutropenia, patients with cystic fibrosis or severe burns and individuals who receive intensive care (Driscoll et al., 2007; Kerr and Snelling, 2009; Worth and Slavin, 2009; Stuart et al., 2010; Rafla and Tredget, 2011).

*P. aeruginosa* intestinal carriage increases from ~3% in normal people to ~20% in hospitalized patients (Stoodley and Thom, 1970). In a case-control study the intestinal colonization by *P. aeruginosa* in cancer patients was 10% before and 31% after hospitalization (Andremont et al., 1989). Studies conducted in oncology–hematology units, found an overall intestinal carriage of *P. aeruginosa* between 11.7 and 37% (Thuong et al., 2003). In another case-control study *P. aeruginosa* intestinal colonization was identified in 17% of controls and 60% of blood cancer patients (Vuotto et al., 2013). These epidemiology data suggest that intestinal colonization by *P. aeruginosa* is prominent among hospitalized cancer patients (Andremont et al., 1989; Vuotto et al., 2013).

The intestinal carriage of *P. aeruginosa* is likely a consequence of the opportunistic nature of this species. Most *P. aeruginosa* infections appear secondary to a breach in host defences. In addition to compromised host immunity, intestinal microbiota play a major role in intestinal defence to infection (Levison, 1973). Thus systemic exposure to antibiotics, which alters intestinal microbiota by reducing the abundance of certain microbes creates the opportunity for intestinal growth of *P. aeruginosa* and other pathogenic bacteria (Hentges et al., 1985).

## Intestinal *P. aeruginosa* as a source of systemic and remote infections

The translocation of endogenous intestinal *P. aeruginosa* extraluminally is an important pathogenic phenomenon and a cause of systemic infections, especially in neutropenic patients with hematological malignancies (Okuda et al., 2010). During the translocation process, bacteria and their products cross the intestinal barrier by traveling between or through the cells of the intestinal epithelium, causing infection and massive inflammation (Alexander et al., 1990; Papoff et al., 2012). Lung infections caused by *P. aeruginosa* are frequent in patients and can occur by direct contamination of the lungs by gastrointestinal flora or through hematogenous spread from the intestine to the lungs. Sepsis and mortality in immunocompromised patients are the results of the presence of highly virulent strains of *P. aeruginosa* within the intestinal tract and the pathogen's ability to adhere to the intestinal epithelial barrier (Marshall et al., 1993; Alverdy et al., 2000; Osmon et al., 2004; Zaborina et al., 2006; Okuda et al., 2010).

*P. aeruginosa* uses different virulence factors that can damage epithelial cells, such as enzymes (proteases and elastases), toxins, adhesins, flagella and protein secretion systems (Sundin et al., 2004). The T3SS enables the injection of at least four effector proteins (ExoS, ExoT, ExoU, and ExoY) into the host cell. ExoS injected into the host epithelial cell migrates to the membrane where it binds to the mammalian factor FXYD3, expressed specifically in the colon and stomach (Okuda et al., 2010). Thus, ExoS may assist the penetration of *P. aeruginosa* through the intestinal epithelial barrier, impairing the defence function of tight junctions against bacterial penetration (Okuda et al., 2010). Moreover, gut inflammation and apoptosis–which can be initiated by the *Pseudomonas* quinolone signal (PQS)–lead to tight junction disruption and an increase of epithelial barrier permeability (Alverdy et al., 2005; An, 2008). Similarly, *P. aeruginosa* lectin PA-I, which is associated with adhesion to epithelial cell layer, is produced after intestinal ischemia and secreted into the intestinal lumen, causing tight junction interruption, epithelial barrier dysfunction and increase of its permeability (Seal et al., 2011).

## Intestinal *P. aeruginosa* exhibits enhanced virulence upon stress, surgery, trauma, and maybe cancer

Cohort studies show that infections are more frequent, severe and lethal among surgical patients (Craven et al., 1988; Sax et al., 2011). Surgical injury can shift the dynamics of the host-pathogen interaction leading to phenotype transformation or phase variation that develops as microbes adapt and respond to novel environments, causing morbidity and mortality (Babrowski et al., 2013). *P. aeruginosa* escalates its virulence and promotes systemic inflammation during various conditions of host stress (Seal et al., 2011). In patients colonized by *P. aeruginosa*, the prolonged surgical injury releases stress-related host factors that can trigger the otherwise dormant colonizers, making them invasive and lethal (Babrowski et al., 2013). Defence mechanisms such as degradative proteases and lipases, exopolysaccharide capsule and outer membrane-derived vesicles (OMVs), which serve as a secretion mechanism for virulence factors, help the pathogen to survive in the host environment (Macdonald and Kuehn, 2013). OMVs are induced in response to physiological stressors and secreted during infection serving multiple roles in bacterial pathogenesis (Macdonald and Kuehn, 2013). In surgically stressed hosts interferon-gamma, endogenous opioids and the hypoxic end-products adenosine and inosine are released into the intestinal lumen where they bind bacteria and activate the expression of PA-I lectin and other virulence factors of *P. aeruginosa.* The PA-I lectin alters the tight junction permeability of the intestinal epithelium to exotoxin A, leading to lethal gut derived sepsis (Long et al., 2008). Moreover, local intestinal depletion of extracellular phosphate (hypophosphatemia), which occurs after surgical injury, can activate virulent pathways due to bacterial sensing of low phosphate, shifting the phenotype of *P. aeruginosa* to that of a lethal strain (Long et al., 2008). Because interferon-gamma, opioids and hypoxia are part of the host response and the therapeutic regiments administered to cancer patients (Dunn et al., 2005; Vaupel and Mayer, 2007), the conditions that accompany cancer may also provide the signals for *P. aeruginosa* virulence induction.

## Can *P. aeruginosa* similarly to other gastrointestinal bacteria facilitate cancer?

Bacteria may initiate oncogenesis because they can induce inflammation and produce cell damaging toxins that facilitate tumorigenesis (Collins et al., 2011; Tjalsma et al., 2012). The characteristic single polar flagella and type 4 pili of *P. aeruginosa* function as initiators of inflammation and adhesins, respectively (Gellatly and Hancock, 2013). *P. aeruginosa* induces Toll-like receptors to activate cytokines, chemokines and COX-2 and recruit cells of the innate and adaptive immune system (Hussain et al., 2003; Holt et al., 2008; de Lima et al., 2012). Epithelial adherence is a property of various bacteria associated with gastrointestinal disease and cancer, such as *Bacteroides fragilis, Streptococcus bovis, Escherichia coli* and *Helicobacter pylori* (Toprak et al., 2006; Selgrad et al., 2008; Abdulamir et al., 2011; Arthur et al., 2012). Cell wall antigens of *S. bovis* induce overexpression of COX-2 and NF-κB *in vitro*, which promote cellular proliferation and angiogenesis (Tafe and Ruoff, 2007; Abdulamir et al., 2009).

E-cadherin, a cell adhesion molecule serves as an antagonist of invasion and metastasis and is found mutated in human carcinomas (Cavallaro and Christofori, 2004; Berx and van Roy, 2009). *B. fragilis* secreted factor BFT cleaves E-cadherin, which is usually bound inside the plasma membrane to β-catenin. The cleavage releases catenin in the cytosol leading to the transcription of the oncogene *c-myc* (Hardy et al., 2000). Similarly, *P. aeruginosa* secreted factor LasI can disrupt adherens junctions and reduce the expression and distribution of E-cadherin and β-catenin in the cell membrane, resulting in changes in cell junction associations and enhanced paracellular permeability (Vikström et al., 2009).

Interestingly, intestinal innate immune responses and stem cells may drive tumor initiation, maintenance and metastasis (Schwitalla et al., 2013). Cancer development is assisted by apoptotic programmed cell death in the tumor microenvironment (Evan and Littlewood, 1998; Lowe et al., 2004; Adams and Cory, 2007) and *P. aeruginosa* uses many virulence factors that induce epithelial cell apoptosis. Intestinal infection with *P. aeruginosa* in *Drosophila* activates the c-Jun N-terminal kinase (JNK) pathway, which causes apoptosis of enterocytes and leads to proliferation of intestinal stem cells (Apidianakis et al., 2009). Importantly, genetic predisposition via an oncogenic form of Ras1/K-Ras oncogene, can synergize with inflammatory signals to induce stem cell-originating tumors characterized by alterations in cell polarity and tissue architecture. Moreover, sustained intestinal infection with *P. aeruginosa* in *Drosophila* induces the Imd/NF-κ B pathway, which synergizes with the oncogene Ras1^*V*12^ to activate the JNK pathway. This leads to invasion and dissemination of oncogenic hindgut cells to distant sites (Bangi et al., 2012; Christofi and Apidianakis, 2013).

## Conclusions

*P. aeruginosa* is a common colonizer of the human intestine upon hospitalization, immunosuppression, antibiotic treatment, surgery, severe trauma and other conditions that cancer patients may face. Not only is *P. aeruginosa* carriage increased in the aforementioned conditions, but also bacteria become more virulent and damaging to the intestinal epithelium upon surgery, injury, and severe stress. Moreover, human isolates of *P. aeruginosa* can induce intestinal pathology and cancer-related epithelial phenotypes in genetically predisposed model hosts. Thus, *P. aeruginosa* appears to have the opportunity and the ability to promote intestinal disease in predisposed hosts, although further proof on the ability of this bacterium to promote tumorigenesis in mammalian models of infection is needed. The lack of epidemiological data linking *P. aeruginosa* to intestinal disease and potentially tumorigenesis in cancer patients may reflect the lack of clinical studies assessing bacterial growth and virulence in relation to cancer recurrence. Because the titter, distribution and virulence of *P. aeruginosa* in the intestine may be very dynamic (Tjalsma et al., 2012), future studies should be designed to repeatedly assess intestinal *P. aeruginosa* abundance and virulence in cancer patients versus healthy individuals. Clinical samples can be assessed for the presence of *P. aeruginosa* via classical microbiology, and next-generation sequencing may offer the chance to assess *P. aeruginosa* transcriptome during infection. Importantly, definite proof of causation of *P. aeruginosa* in morbidity and mortality of cancer patients can only be achieved if targeted elimination of *P. aeruginosa* from these patients improves the outcome of their disease. In Figure 1 we illustrate a roadmap to specifically assess the role of *P. aeruginosa* in intestinal disease and tumorigenesis. It is conceivable that similar principles can be used to assess causality between intestinal disease and many other opportunistic pathogens harbored by the human gut.
